# Liraglutide ameliorates myocardial damage in experimental diabetic rats by inhibiting pyroptosis via Sirt1/AMPK signaling 

**DOI:** 10.22038/IJBMS.2021.56771.12677

**Published:** 2021-10

**Authors:** Zhe Zhang, Xing Wang, Linlin Yang, Linquan Yang, Huijuan Ma

**Affiliations:** 1 Hebei Key Laboratory of Metabolic Diseases, Hebei General Hospital, Shijiazhuang 050051, P.R. China; 2 Department of Endocrinology, Hebei General Hospital, Shijiazhuang 050051, P.R. China

**Keywords:** Cardiovascular disease, Diabetes mellitus, Inflammasomes, Rats, Sirtuin 1

## Abstract

**Objective(s):**

Liraglutide, a well-established drug for treating diabetes mellitus (DM), has recently gained attention for its cardiovascular benefits in diabetes via multiple cellular activities; however, whether liraglutide improves myocardial damage by inhibiting pyroptosis and the mechanisms of these potential effects remain unknown.

**Materials and Methods:**

In this study, high-fat diet feeding and low-dose streptozotocin (STZ) injection were used to construct a rat DM model. Rats with fasting blood glucose (FBG) levels >16.7 mmol/l received subcutaneous injections of liraglutide (0.2 mg/kg) for 4 weeks. Metabolic parameters, the heart weight/body weight (HW/BW) ratio, and histopathology were examined. Protein levels of inflammatory, pyroptosis, and NOD-like receptor protein 3 (NLRP3) inflammasome markers were assessed via Western blotting. In *in vitro* studies, a sirtuin 1 (Sirt1) inhibitor (EX 527, 200 nM) and an AMP-activated protein kinase (AMPK) inhibitor (compound C, 20 µM) were used to inhibit Sirt1 and AMPK pathways, respectively.

**Results:**

Liraglutide significantly attenuated cardiac hypertrophy, pathological changes, inflammation, pyroptosis, and NLRP3 inflammasome activation, accompanied by increased Sirt1 and AMPK activation. Consistent with the *in vivo* results, liraglutide attenuated high glucose (HG)-induced pyroptosis and NLRP3 inflammasome activation along with enhanced Sirt1 and AMPK activation. After blockade of Sirt1 and AMPK signaling, the protective effect of liraglutide was restrained. Notably, EX 527 abolished the stimulatory effect of liraglutide on Sirt1 and AMPK signaling, whereas compound C blunted AMPK signaling without affecting Sirt1 signaling.

**Conclusion:**

Liraglutide may protect against myocardial damage by activating the Sirt1/AMPK signaling pathways to inhibit cellular pyroptosis in DM.

## Introduction

Diabetes mellitus (DM) has become a common and severe disease characterized by high morbidity and mortality worldwide. Clinical and preclinical studies demonstrated that DM increases the incidence of cardiovascular complications (e.g., myocardial infarction, ischemic stroke, heart failure, and peripheral artery disease), increasing the risk of cardiac death (1). The features of diabetic cardiomyopathy (DCM) include cardiac hypertrophy, which comprises structural and functional abnormalities that are independent of coexisting hypertension, obesity, and coronary artery disease. The pathogenic mechanisms of DM and its complications involve glycolipid metabolic disorder, oxidative stress, inflammation, mitochondrial dysfunction, calcium overload, lipotoxicity, and other processes (2-4). However, the underlying molecular mechanisms of DCM remain unclear. Beyond this, there is currently no safe and effective strategy for treating DCM in the clinic, and its incidence and mortality rates remain high. 

Numerous studies illustrated that inflammasomes participate in the pathological progression of DM, and they are novel drug targets for improving the outcomes of DM and its cardiovascular complications (5-7). Among the inflammasomes, NOD-like receptor protein 3 (NLRP3) is the major and ubiquitously identified NOD-like receptor gene. In response to various stimuli, NLRP3 binds to its adaptor ASC, followed by the activation of pro-caspase-1, which facilitates the cleavage and maturation of the inflammatory cytokines interleukin (IL)-1β and IL-18, thus causing a newly described form of necrosis (pyroptosis). Emerging studies have demonstrated that NLRP3 overactivation is involved in the progression of DCM. Importantly, NLRP3 inflammasome silencing exerts a protective effect against DCM in diabetic rat models and high glucose (HG)-treated H9c2 cardiomyocytes (8-10). 

The activity of sirtuin 1 (Sirt1), a nicotinamide adenine dinucleotide-dependent histone deacetylase in mammalian cells, has been associated with many biological activities such as cell differentiation, apoptosis, autophagy, energy metabolism, and protection against DNA damage. Sirt1 is known to protect the pathogenesis of DCM along with regulation of mitochondrial biogenesis, metabolic signaling pathways, and pro-inflammatory pathways. In the diabetic heart, Sirt1 suppresses nuclear factor (NF)-κB activation by deacetylating the p65 subunit of the NF-κB complex, thereby blunting the expression of inflammatory factors (11). Moreover, Sirt1 was found to ameliorate hyperglycemia-induced oxidative stress and inflammation by intensifying the Sirt1/peroxisome proliferator-activated receptor-γ coactivator (PGC)-1α signaling pathway (12). Recently, the regulatory effect of Sirt1 on NLRP3 inflammasome has gained much attention. A study demonstrated that Sirt1 inhibits hyperglycemia-induced renal inflammation by negatively regulating the NLRP3 inflammasome in DM (13). Sirt1-dependent inhibition of NLRP3 inflammasome activation was also revealed to be involved in HG-induced diabetic endothelial dysfunction (14). These studies implicate the involvement of Sirt1 in DCM through inhibition of the NLRP3 inflammasome. AMP-activated protein kinase (AMPK), a key downstream target of Sirt1, shares commonalities with Sirt1 in metabolism and cellular survival (15, 16). Activation of AMPK has been demonstrated to confer a solid protective effect against diabetic myocardial injury (17). Hence, targeting Sirt1/AMPK signaling may be a potential therapeutic strategy in DCM. However, studies illustrating the specific relationship of Sirt1 with AMPK signaling in DM-injured diabetic myocardium are limited.

liraglutide (LRG) is a therapeutic drug for treating DM that has become increasingly popular because of its cardiovascular benefits. Researchers have suggested that LRG exerts its cardioprotective effects in rodents and humans via anti-inflammatory, anti-oxidant, and anti-atherosclerosis mechanisms (18, 19). However, whether LRG regulates cardiomyocyte pyroptosis under diabetic conditions and the underlying cellular mechanisms of such an effect remain elusive. This study sought to clarify whether LRG inhibits myocardial pyroptosis by ameliorating activation of the NLRP3 inflammasome via Sirt1/AMPK signaling using a diabetic rat model *in vivo* and an H9c2 cell line *in vitro*.

## Materials and Methods

***Animals models***

This study was approved by the Institutional Ethical Committee of Hebei General Hospital. Six-week-old male Sprague-Dawley rats (weight, 200-220 g), provided by Vital River Laboratories Co., Ltd. (Beijing, China, SCXK-2016-0006), were housed under standard conditions (20–22 ^°^C; 12-hr/12-hr light/dark cycle). All rats were fed adaptively for 1 week with food and water *ad libitum* and were assigned to three groups: normal control (NC) group, DM group, and DM+LRG group (n=10 in each group). The NC group was fed a standard rodent diet throughout the experiment, whereas the other two groups were fed a high-fat diet (HFD) containing 60% kcal fat, 20% kcal carbohydrates, and 20% kcal protein for 8 weeks. After 8 weeks of HFD feeding, rats in the DM and DM+LRG groups received an intraperitoneal injection of 30 mg/kg STZ (Solarbio, Beijing, China) (20), whereas rats in the NC group were injected with the same dose of citrate buffer. At 72 hr following STZ treatment, blood samples were obtained from the rat tail vein after 12 hr of fasting, and the glucose concentration was examined using an automatic analyzer (Roche Diagnostics, Indianapolis, IN, USA). Rats with fasting blood glucose (FBG) levels >16.7 mmol/l were considered diabetic. After 1 week, rats in the DM+LRG group were administered 0.2 mg/kg LRG (Novo Nordisk, Denmark) via subcutaneous injection once daily (20). The NC and DM groups received the same volume of normal saline. All rats were maintained for 4 weeks.

***Blood parameters***

After treatment with LRG, blood was obtained from the abdominal aorta of each rat under anesthesia with pentobarbital sodium (30 mg/kg, IP) and centrifuged at 1500 g for 15 min at 4 ^°^C. Serum triglyceride (TG), total cholesterol (TC), low-density lipoprotein cholesterol (LDL-C), high-density lipoprotein cholesterol (HDL-C), creatine kinase (CK), and lactate dehydrogenase (LDH) levels were measured using detection kits (Jiancheng, Nanjing, China). 

***Measurement of the heart weight/body weight (HW/BW) ratio***

Finally, rats were euthanized via decapitation under anesthesia, and their hearts were immediately harvested, washed with PBS, superficially blotted, and weighed. The myocardial hypertrophy was determined by estimating the HW/BW ratio.

***Hematoxylin-eosin (H&E) assay***

The left ventricular tissue sections were fixed in 4% formalin, and paraffin-embedded sections (5 µm) were stained separately with H&E. The histopathological characteristics of the hearts were observed using a light microscope (magnification: ×400).

***TUNEL staining***

Cell death in the heart tissues was determined using a TUNEL staining kit (Roche, Mannheim, Germany). Images were captured using fluorescence microscopy (Nikon Instech Co. Ltd., Tokyo, Japan), and nuclei labeled with DAPI and TUNEL were considered TUNEL-positive. The ratio of TUNEL-positive myocytes (green) to the total number of myocytes (blue) was used to calculate the percentage of pyroptotic cells.

***Cell culture and experimental protocol***

H9c2 cardiomyocytes were cultured with Dulbecco modified Eagle’s medium-F12 with Ham’s nutrition mixture (Gibco, USA) containing 10% fetal bovine serum (Gibco) in a humidified atmosphere of 95% air and 5% CO_2_ at 37 ^°^C. After reaching 80% confluence, cells were treated with 5.5 mM glucose (normal glucose [NG]) or 33.3 mM glucose (HG) (10) in the absence or presence of 100 nM LRG (21) for 48 hr. To inhibit Sirt1 and AMPK signaling, cells were pretreated with 200 nM EX 527 and 20 µM compound C (Selleck Chemicals, Houston, USA), respectively, for 24 hr before HG stimulation as described previously (14, 18).

***Cell viability***

To assess cell viability, a CCK-8 assay kit (Dojindo Laboratories, Kyushu, Japan) was used. Cells were seeded in 96-well plates (8×10^3^ cells per well) and incubated with CCK-8 solution (1:10 dilution) after the aforementioned treatments. Then, we detected the absorbance at 450 nm using a microplate reader (Thermo Fisher Scientific, CA, USA), and the values were normalized to those in the NG group. 

***Flow cytometry***

After the cardiomyocytes reached confluence, cell death was assessed using an apoptosis assay kit (Multisciences Biotechnology, Hangzhou, China). H9c2 cells were stained with 5 µl of Annexin-V FITC and 10 µl of propidium iodide (PI), and flow cytometry (Beckman Coulter, CA, USA) was used to measure pyroptotic cells.

***Western blot analysis***

Total protein was extracted from frozen hearts or H9c2 cells, and the total protein level was detected using a BCA assay kit (Thermo Fisher Scientific). Proteins were separated by electrophoresis and transferred to a PVDF membrane. Then, the membrane was incubated with specific primary antibodies and secondary antibodies. Antibodies against the atrial natriuretic peptide (ANP, 1:600) and the fetal isoform of myosin heavy chain (β-MHC, 1:600) were obtained from Santa Cruz (CA, USA). Antibodies against cleaved caspase-3 (1:1000), cleaved caspase-8 (1:1000), cleaved caspase-9 (1:1000), Sirt1 (1:1000), AMPK (1:1000), and p-AMPK (1:1000) were purchased from CST (Danvers, MA, USA). Antibodies against IL-6 (1:1000), tumor necrosis factor (TNF)–α (1:500), NLRP3 (1:1000), ASC (1:2000), caspase-1 (1:500), IL-1β (1:1000), and IL-18 (1:1000) were procured from Abcam (Cambridge, UK). Signals were semi-quantified with a chemiluminescence system using Image J software (version 1.48; National Institutes of Health, Bethesda, MD, USA).

***Statistical analysis***

Data are presented as the mean±standard error of the mean (SEM), and statistical analyses were performed using SPSS 18.0 software. One-way ANOVA followed by Fisher’s protected least significant difference test or Student’s *t*-test was conducted to test significance between different groups. *P*-value <0.05 denoted statistical significance. 

## Results

***LRG ameliorates metabolic deformity in diabetic rats***

As presented in Table 1, FBG levels in the DM and DM+LRG groups were significantly higher than those in the NC group. However, FBG levels were lower in the DM+LRG group than in the DM group. In addition, increased serum TC, TG, and LDL-C levels and reduced HDL-C levels were observed in the DM group. The administration of LRG normalized all of these lipid parameters. These results suggested that the diabetic rat model was successfully constructed, and LRG administration was effective in controlling glucose and lipids. 

***LRG attenuates myocardial tissue injury in diabetic rats***

**Figure 1:**
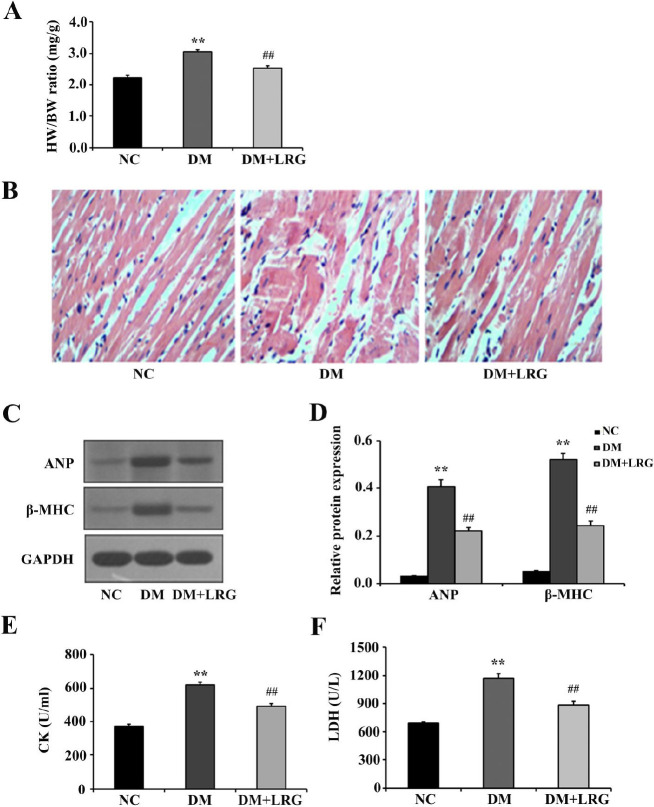
Liraglutide attenuates myocardial tissue injury in diabetic rats. (A) Quantitative da-ta for the heart weight/body weight (HW/BW) ratio; (B) Representative images of myocar-dium tissue sections stained with hematoxylin-eosin (×400); (C) Representative images of Western blotting; (D) Atrial natriuretic peptide (ANP) and fetal isoform of myosin heavy chain (β-MHC) expression; (E) Serum creatine kinase (CK) levels; (F) Serum lactate dehy-drogenase (LDH) levels. Data are presented as the mean±SEM, n=5–10 per group. ^**^P<0.01 vs NC; ^##^*P<*0.01 vs DM

NC: normal control; DM: diabetes mellitus; LRG: liraglutide

Diabetic-induced myocardial damage was followed by adverse myocardial remodeling; therefore, we investigated cardiac hypertrophy in diabetic rats. As shown in Figure 1A, the HW/BW ratio was increased in the DM group, and LRG treatment reduced the hyperglycemia-induced increase of the HW/BW ratio. Then, we examined the morphological and histological features of heart tissues using H&E staining. Compared with the NC rats, DM rats’ hearts displayed structural abnormalities, but the disordered diabetic cardiac muscle fibers were repaired by LRG (Figure 1B). Western blot analysis confirmed that protein expression of ANP and β-MHC, two markers of cardiac hypertrophy, was increased in the DM group, and these increases were all reduced by LRG (Figures 1C, D). Additionally, serum CK and LDH activities are usually regarded as markers of damage to cardiac structural integrity in diabetic rats. In the present study, diabetic rats displayed increased CK and LDH release; however, LRG treatment markedly decreased the levels of both biochemical markers of myocardial damage (Figures 1E, F). These results suggested a potential effect of LRG against myocardial tissue injury in diabetic rats. 

***LRG reduces myocardial inflammation and pyroptosis in diabetic rats***

**Figure 2:**
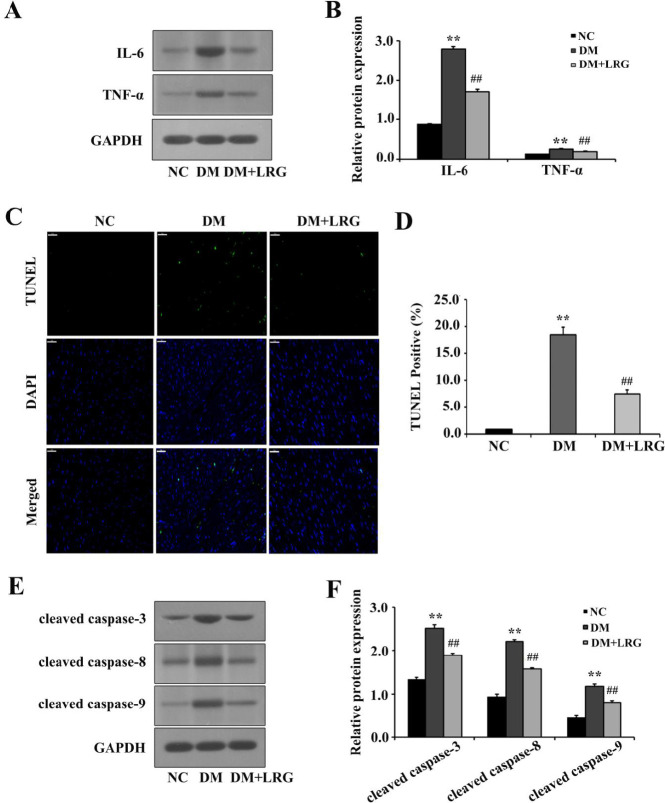
Liraglutide reduces myocardial inflammation and pyroptosis in diabetic rats. (A) Representa-tive images of Western blotting; (B) Interleukin (IL)-6 and tumor necrosis factor (TNF)-α ex-pression; (C) Representative images of myocardium tissue sections stained with TUNEL (×400, bar=50 µm). The pyroptotic cells were detected via TUNEL staining (green), and the nuclei were detected using DAPI (blue); (D) Quantitative data for the TUNEL-positive my-ocytes; (E) Representative images of Western blotting; (F) Cleaved caspase-3, cleaved caspa-se-8, and cleaved caspase-9 expression. Data are presented as the mean±SEM, n=5–10 per group. ^**^P<0.01 vs NC; ^##^*P<*0.01 vs DM

NC: normal control; DM: diabetes mellitus; LRG: liraglutide

Diabetes-induced cardiac hypertrophy is reported to be highly related to inflammatory response and pyroptosis. First, we examined cardiac inflammation by detecting the protein expression of inflammation factors. Enhanced expression of IL-6 and TNF-α was identified in the DM group compared with those in the NC group. However, these alterations were reversed by LRG treatment (Figures 2A, B). Then, TUNEL assay revealed that LRG reduced the percentage of TUNEL-positive cells (Figures 2C, D). Coincident with cardiac pyroptosis, Western blot analysis also furnished evidence that LRG down-regulated the expression of pyroptosis markers, including cleaved caspase-3, cleaved caspase-8, and cleaved caspase-9 (Figures 2E, F). These results indicated that the inhibition of cardiac hypertrophy by LRG may be potentially attributed to its anti-inflammatory and anti-pyroptotic properties.

***LRG down-regulates NLRP3 inflammasome expression in diabetic rats***

**Figure 3:**
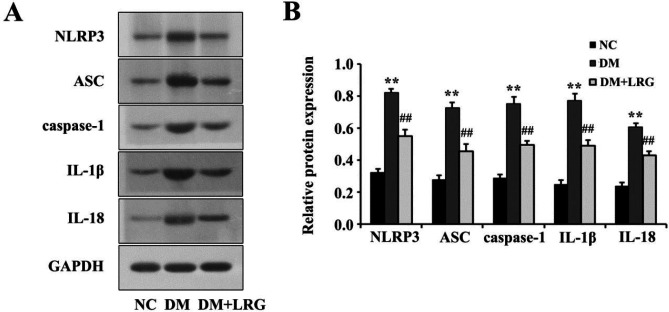
Liraglutide down-regulates NOD-like receptor protein 3 (NLRP3) inflammasome expression in diabetic rats. (A) Representative images of Western blotting; (B) NLRP3 expression. Data are presented as the mean±SEM, n=5 per group. ^**^P<0.01 vs NC; ^##^*P<*0.01 vs DM

NC: normal control; DM: diabetes mellitus; LRG: liraglutide

The NLRP3 inflammasome triggers the inflammatory response by releasing mature cytokines (e.g., IL-1β and IL-18) and participates in DM-induced myocardial injury. To gain insights into the downstream signaling mechanisms through which LRG mediates pyroptosis, we assessed the effects of LRG on cardiac NLRP3 inflammasome expression via Western blotting. In comparison with the NC group levels, rats in the DM group exhibited elevated expression of myocardial NLRP3, ASC, caspase-1, IL-1β, and IL-18, whereas LRG treatment reduced their expression (Figures 3A, B). 

***LRG activates myocardial Sirt1 and AMPK signaling in diabetic rats***

**Figure 4:**
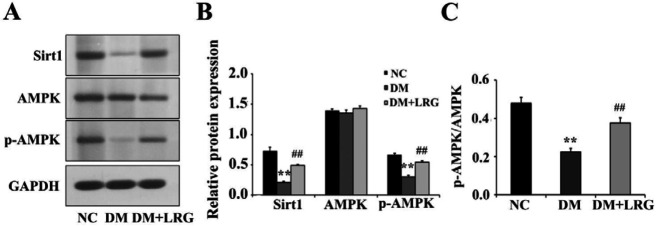
Liraglutide activates myocardial sirtuin 1 (Sirt1) and AMP-activated protein kinase (AMPK) signaling in diabetic rats. (A) Representative images of Western blotting; (B) Sirt1, AMPK, and p-AMPK expression; (C) p-AMPK/AMPK ratio. Data are presented as the mean±SEM, n=5 per group. ^**^P<0.01 vs NC; ^##^*P<*0.01 vs DM

NC: normal control; DM: diabetes mellitus; LRG: liraglutide

Previous studies demonstrated that Sirt1 and AMPK signaling pathways serve crucial roles in the apoptotic effect of LRG in STZ-induced diabetic rats’ hearts. Hence, we evaluated the myocardial expression of Sirt1 and AMPK. As shown in our data, significant decreases in the protein expression of Sirt1 and the p-AMPK/AMPK ratio were observed in diabetic rats, and these decreases were reversed by LRG treatment (Figures 4A-C). These results suggested that Sirt1 and AMPK signaling contributed greatly to the cardioprotective effects of LRG.

***LRG alleviates pyroptosis via Sirt1 and AMPK signaling in HG-induced H9c2 cells***

**Figure 5:**
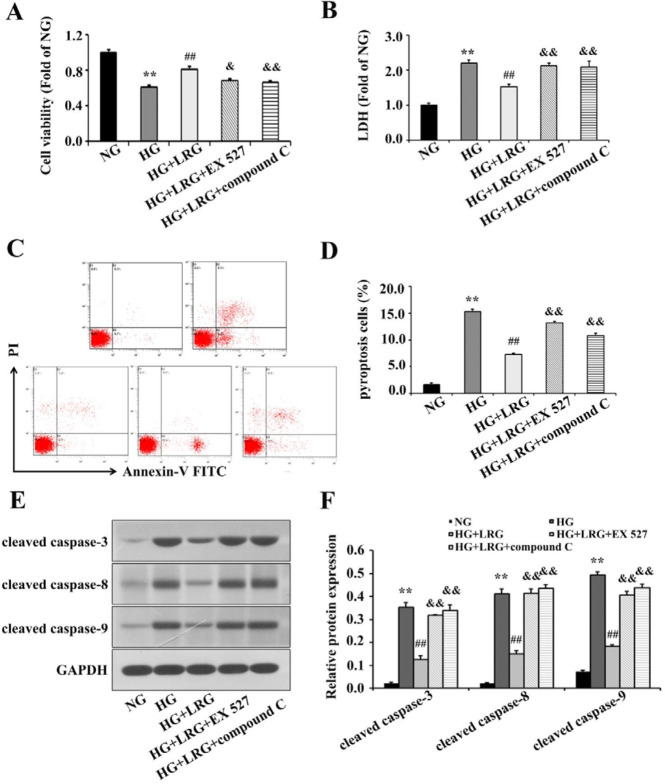
Liraglutide alleviates pyroptosis via sirtuin 1 (Sirt1) and AMP-activated protein kinase (AMPK) signaling in HG-treated H9c2 cells. (A) Cell viability was measured using a CCK-8 kit; (B) Lactate dehydrogenase (LDH) release was measured using a cytotoxicity detection LDH kit; (C) Representative images of flow cytometry of H9c2 cells; (D) Quantitative analy-sis of pyroptotic cells; (E) Representative images of Western blotting; (F) Cleaved caspase-3, cleaved caspase-8, and cleaved caspase-9 expression. Data are presented as the mean±SEM, n=3 per group. ^**^P<0.01 vs NG; ^##^*P<*0.01 vs HG; ^&^*P<*0.05, ^&&^*P<*0.01 vs HG+LRG

NG: normal glucose; HG: high glucose; LRG: liraglutide

To examine the cardioprotective effects of LRG on DM hearts, cultured H9c2 cells exposed to HG stimulation were used. As shown in Figures 5A–D, HG dramatically decreased cell viability and increased LDH release and the percentage of annexin V/PI-positive H9c2 cells compared with the findings in NG-treated cells, whereas pretreatment with 100 nM LRG significantly reduced HG-induced cell death. Meanwhile, LRG treatment also exerted anti-pyroptotic effects on H9c2 cells and prevented the up-regulation of pyroptosis-related proteins (Figures 5E, F). Mechanistically, inhibition of Sirt1 and AMPK by the specific antagonists EX 527 (200 nM) and compound C (20 µM), respectively, decreased cell viability and restricted the anti-pyroptotic activities of LRG (Figures 5A-F). Treatment with neither EX 527 nor compound C affected cell viability or pyroptosis signaling under the present experimental concentrations (data not shown). These data suggested that LRG may alleviate HG-induced cardiomyocytes injury, at least partially, via Sirt1 and AMPK signaling.

***LRG inhibits activation of the NLRP3 inflammasome via Sirt1 and AMPK signaling in HG-induced H9c2 cells***

**Figure 6:**
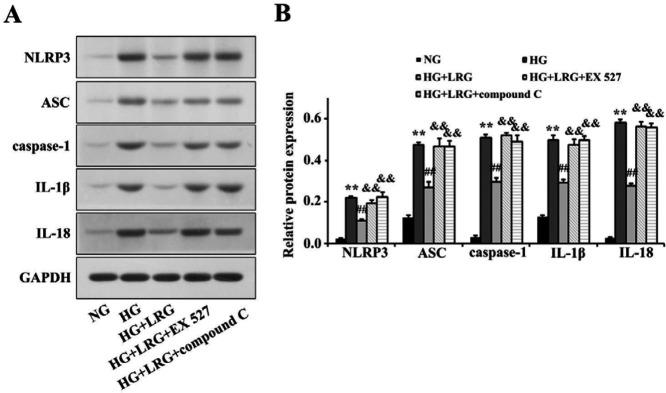
Liraglutide inhibits activation of the NOD-like receptor protein 3 (NLRP3) inflammasome via sirtuin 1 (Sirt1) and AMP-activated protein kinase (AMPK) signaling in HG-treated H9c2 cells. (A) Representative images of Western blotting; (B) NLRP3 expression. Data are pre-sented as the mean±SEM, n=3 per group. ^**^P<0.01 vs NG; ^##^*P<*0.01 vs HG; ^&&^*P<*0.01 vs HG+LRG

NG: normal glucose; HG: high glucose; LRG: liraglutide

Western blotting revealed that HG notably promoted NLRP3, ASC, caspase-1, IL-1β, and IL-18 expression in H9c2 cells, and their expression was attenuated by LRG pretreatment. However, the inhibitory effects of LRG on the NLRP3 inflammasome were ablated by EX 527 and compound C. Our results indicated that HG dramatically suppressed NLRP3 inflammasome activation via modulating Sirt1 and AMPK signaling (Figures 6A, B). 

***LRG-induced activation of AMPK signaling is mediated via the upstream kinase Sirt1 in HG-induced H9c2 cells***

**Figure 7:**
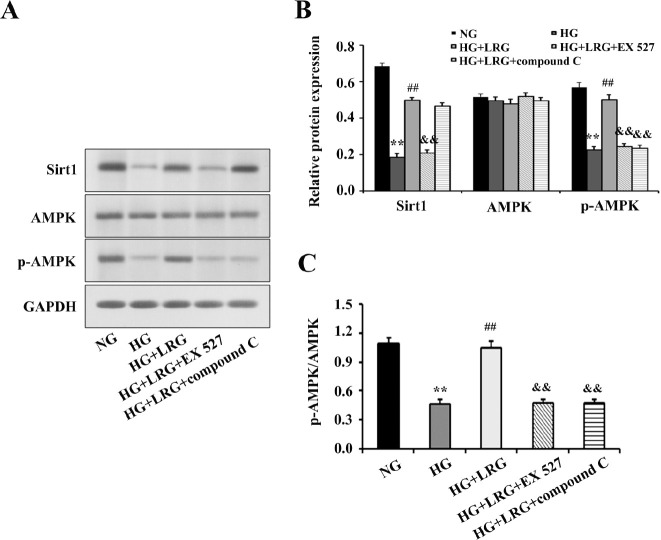
EX 527 blunted cellular sirtuin 1 (Sirt1) and AMP activated protein kinase (AMPK) signaling whereas compound C reduced p-AMPK expression without affecting Sirt1 signaling. (A) Representative images of Western blotting; (B) Sirt1, AMPK, and p-AMPK expression; (C) p-AMPK/AMPK ratio. Data are presented as the mean±SEM, n=3 per group. ^**^P<0.01 vs NG; ^##^*P<*0.01 vs HG; ^&&^*P<*0.01 vs HG+LRG

NG: normal glucose; HG: high glucose; LRG: liraglutide

To clarify the relationship between Sirt1 and AMPK concerning the cardioprotective effects of LRG, we directly assessed the protein expression of Sirt1 and AMPK. As shown in Figures 7A-C, HG treatment significantly decreased the protein expression of Sirt1 and the p-AMPK/AMPK ratio compared with the findings in NG-treated cells. However, pretreatment with 100 nM LRG significantly increased Sirt1 protein expression and the p-AMPK/AMPK ratio. In addition, we found that EX 527 blunted cellular Sirt1 and AMPK signaling by decreasing the protein expression of Sirt1 and p-AMPK (compared with the findings in the HG+LRG group), whereas compound C blocked AMPK expression without changing Sirt1 expression. These data suggested that Sirt1 may act upstream of AMPK signaling in mediating the cardioprotective effect of LRG in DM.

**Table 1:** Metabolic characteristics of the experimental animals

Group	FBG (mmol/L)	TG (mmol/L)	TC (mmol/L)	HDL-C (mmol/L)	LDL-C (mmol/L)
NC	5.95±**0.23**	0.71±**0.06**	1.49±**0.09**	0.57±**0.02**	0.29±**0.01**
DM	18.22±0.45**	1.64±0.07**	2.75±0.09**	0.31±0.01**	0.42±0.02**
DM+LRG	13.29±0.62**^#^	1.22±0.08**^##^	2.15±0.04**^##^	0.42±0.02**^##^	0.37±0.01**^##^

Data are presented as the mean±SEM. n=8–10 per group. ^**^P<0.01 vs NC; ^#^*P<*0.05, ^##^*P<*0.01 vs DM

NC: normal control; DM: diabetes mellitus; LRG: liraglutide; FBG: fasting blood glucose; TG: triglyceride; TC: total cholesterol; HDL-C: high-density lipoprotein cho-lesterol; LDL-C, low-density lipoprotein cholesterol

## Discussion

LRG, a novel GLP-1 receptor agonist, has been approved for the treatment of DM. Recently, the use of LRG for treating cardiovascular diseases has attracted increasing interest, and this cardioprotective effect is independent of its hypoglycemic action (22). LEADER trial data revealed a lower rate of hospitalization for congestive heart failure and lower risks of cardiovascular mortality and all-cause death among patients with DM on LRG therapy (23). These beneficial actions of LRG may facilitate its beneficial effects on DM-associated cardiovascular complications, and the use of LRG may provide further clinical benefits for patients with DCM. However, little evidence is available to explain the mechanism underlying these effects. In the present study, a DCM rat model was successfully established, and its metabolic and structural characteristics were consistent with those described in previous studies (20, 24). Moreover, we found that irrespective of the cause, treatment with LRG for 4 weeks alleviated myocardial remodeling and pyroptosis, thus restoring the metabolic homeostasis of cardiomyocytes. These observations may explain the protective effect of LRG against DCM. Mechanistically, the *in vivo *and* in vitro* data of the present study indicated that Sirt1 and its downstream target AMPK were involved. Our study for the first time described a potential relationship between Sirt1 and AMPK signaling regarding LRG-mediated anti-inflammatory and anti-pyroptotic effects in the DM setting.

Emerging evidence suggests that hyperglycemia-induced oxidative stress triggers myocardial inflammation in diabetes (3). Myocardial inflammation plays a critical role in the pathogenesis of DCM, which causes heart failure and DCM by triggering apoptosis, fibrosis, and myocardial remodeling in human and animal models of diabetes. Recently, evidence points to NLRP3 overactivation coupled to inflammatory response and cell death pathways as primary etiologic factors in the DCM setting. Hyperglycemia can trigger NLRP3 overactivation and promote the production of cleaved caspase-1, thus accelerating the maturation and release of IL-1β and IL-18, which induce pyroptosis and finally lead to cardiac dysfunction and heart failure (25). Moreover, NLRP3 gene silencing *in vivo* and *in vitro* markedly improved cardiac inflammation, pyroptosis, fibrosis, and cardiac function, which were paralleled by decreased expression of pro-inflammatory cytokines and cardiac cell death (10). In this study, we demonstrated that inflammation and pyroptosis perturbed glycolipid metabolism in cardiomyocytes and induced myocardial damage in accordance with previous findings (26). Our *in vitro *data also illustrated that HG-treated H9c2 cells displayed the typical characteristics of pyroptosis. More recently, Chen *et al*. demonstrated that LRG attenuated TNF-α– and hypoxia-induced H9c2 cells injury by inhibiting the NLRP3 inflammasome pathway (27). Consistently, we unveiled the anti-pyroptotic role of LRG both *in vivo *(Figure 2) and *in vitro* (Figure 5). Specifically, cellular injury and the expression of pyroptosis-related proteins and NLRP3 complex in the myocardium and H9c2 cells were markedly attenuated by LRG. These data indicated that the NLRP3 inflammasome might be a key mechanistic and treatment target for DCM. 

Sirt1 is a member of the highly conserved class III histone deacetylase family, which has been implicated in glucose homeostasis and cardiac protection through a complex signaling network. Sirt1 suppression has been characterized as a contributing factor in metabolic disorders and various cardiovascular diseases (28). Sirt1 expression was shown to be reduced in diabetic hearts and resulted in insulin resistance, mitochondrial dysfunction, and impaired antioxidant defenses (12). Recent studies have demonstrated that cardiac-specific knockdown of Sirt1 contributed to causing phenotypes resembling DCM (29, 30). Thus, targeting Sirt1 may be a promising treatment strategy within a diabetic setting. Researchers confirmed the crucial positive effects of Sirt1 activation to alleviate oxidative stress and inflammation, promote autophagy, and prevent myocardial dysfunction in diabetic hearts and HG-treated H9c2 cells (31, 32). In agreement with these findings, experiments using our *in vivo *and* in vitro* models also uncovered reduced myocardial Sirt1 expression, and treatment with LRG abrogated these effects. Of note, the* in vitro* experiments further proved that LRG-mediated inhibition of NLRP3 inflammation was Sirt1 dependent. Consistent with our results, a study showed that gene silencing of Sirt1 abolished the inhibitory effect of Sirt1 activator on NLRP3 inflammasome activation in vascular endothelial cells under the stimulation of LPS and ATP (33). Yao *et al*. also reported that Sirt1 inhibitor sirtinol enhanced the NLRP3 inflammasome activation and oxidative stress in DCM model mice (34). 

Our study further illustrated that AMPK-dependent pathways could be the downstream targets of Sirt1 signaling in mediating the cardioprotective role of LRG. In fact, as an important metabolic sensor, AMPK itself plays a crucial role in regulating fatty acid and glucose metabolism, autophagy, endoplasmic reticulum stress, and cardioprotective protein expression (35). Intriguingly, AMPK activation has been verified to confer cardioprotection against myocardial injury in DCM by attenuating pyroptosis. Study showed that Exendin-4, a GLP analog, protected against hyperglycemia-induced cardiomyocyte pyroptosis via the AMPK/thioredoxin-interacting protein (TXNIP) pathway (36). Additionally, AMPK/mTOR signaling was demonstrated to play a crucial part in metformin-induced inhibition of NLRP3 inflammation in DCM (37). In the present study, we found that LRG administration markedly increased p-AMPK protein expression in DM hearts and H9c2 cells following HG stimulation**. **However, the AMPK inhibitor compound C, not only blunted AMPK activation but also blocked the anti-pyroptotic and cardioprotective effect of LRG. 

Currently, conflicting results regarding the relationship between Sirt1 and AMPK signaling under pathological conditions have emerged from recent studies. Liu *et al*. have shown that AMPK may be a pivotal upstream regulator of Sirt1 and activation of AMPK by Sirt1 plays an essential role in glucose metabolism and neuronal apoptosis, and exerts protection against myocardial hypertrophy and cognition dysfunction (38, 39). However, some evidence also demonstrated that Sirt1 markedly increased the phosphorylation levels of AMPK in the diabetic state. For instance, a study provided evidence that the AMPK/mTOR axis acted as a downstream target of Sirt1 and prevented HG-induced endothelial death and dysfunction (40). Another study found that vitamin D prevented oxidative stress and up-regulated glucose uptake via Sirt1/AMPK/IRS1/GLUT4 cascade in HG-treated adipocytes and adipose tissue of diabetic mice (41). Significantly, our *in vitro *experiment found that co-treatment with the Sirt1 inhibitor EX 527 markedly abrogated the effects of LRG on Sirt1 and AMPK signaling; however, the AMPK inhibitor compound C significantly blunted AMPK signaling without changing Sirt1 expression. Our results indicated that AMPK signaling was activated via Sirt1 in the protective mechanism of LRG against DM-induced myocardial damage. The lack of agreement among different experiments is likely attributable to the complexity of the multiple components of Sirt1/AMPK signaling, the cellular context, and the type of stimuli studied. 

We and others uncovered the potential beneficial effects of LRG therapy in protecting against myocardial damage in the diabetic state by attenuating myocardial cell pyroptosis. There were some limitations in this study. First, to confirm the direct benefit of LRG on DM-induced myocardial damage, the LRG-only treated group should be selected both in *in vivo *and *in vitro* studies. In addition, although we demonstrated that Sirt1/AMPK signaling was activated following LRG administration, the mechanism by which Sirt1 activation stimulates AMPK remains relatively obscure. Because Sirt1 participates in multiple biological processes through deacetylation of its targets, we are tempted to speculate that alteration of acetylation and deacetylation activity may be involved. Mechanistically, Sirt1 decreases the lysine acetylation of LKB1, leading to the activation of AMPK, which confers cardioprotection by directly phosphorylating its target proteins (e.g., ULK1 and PGC-1α) or via transcriptional regulation of its target genes (e.g., Akt, mTOR, Foxo, and p300) (42). Further study is needed to decipher the underlying mechanisms through which LRG protects against DM-exacerbated myocardial damage in rats. 

## Conclusion

The present study demonstrated that LRG treatment can protect against myocardial injury in the diabetic state by inhibiting NLRP3 inflammasome-dependent inflammation and pyroptosis. More importantly, Sirt1/AMPK signaling mediates, at least partially, the cardioprotective effects of LRG. This should be considered in future studies evaluating LRG as a potential novel drug for treating diabetes-related cardiovascular disease. Activation of Sirt1/AMPK pathways can be targeted to protect cardiomyocyte survival *via* yet unknown mechanisms.

## Authors’ Contributions

ZZ and XW performed the study. ZZ wrote the manuscript. XW prepared figures; Linlin Yang contributed to data analysis. Linquan Yang contributed materials and analysis tools. HM approved final version of manuscript.

## Conflicts of Interest

No conflicts of interest have been declared by the authors.
